# Acetone Prospect as an Additive to Allow the Use of Castor and Sunflower Oils as Drop-In Biofuels in Diesel/Acetone/Vegetable Oil Triple Blends for Application in Diesel Engines

**DOI:** 10.3390/molecules25122935

**Published:** 2020-06-25

**Authors:** Laura Aguado-Deblas, Jesus Hidalgo-Carrillo, Felipa M. Bautista, Diego Luna, Carlos Luna, Juan Calero, Alejandro Posadillo, Antonio A. Romero, Rafael Estevez

**Affiliations:** 1 Departamento de Química Orgánica, Universidad de Córdoba, Campus de Rabanales, Ed. Marie Curie, 14014 Córdoba, Spain; aguadolaura8@gmail.com (L.A.-D.); Jesus.hidalgo@uco.es (J.H.-C.); qo1baruf@uco.es (F.M.B.); qo2luduc@uco.es (C.L.); p72camaj@gmail.com (J.C.); qo1rorea@uco.es (A.A.R.); q72estor@uco.es (R.E.); 2 Seneca Green Catalyst S.L., Campus de Rabanales, 14014 Córdoba, Spain; seneca@uco.es

**Keywords:** acetone, castor oil, sunflower oil, biofuel, diesel engine, smoke opacity, Bosch smoke number

## Abstract

The present paper investigates the feasibility of using acetone (ACE) in triple blends with fossil diesel (D) and straight vegetable oils (SVOs) as alternative fuel for diesel engines. In this respect, ACE is selected as an oxygenated additivedue to its favorable propertiesto be mixed with vegetable oils and fossil diesel. In fact, the very low kinematic viscosity allows reduces the high viscosity of SVOs. ACE’s oxygen content, low autoignition temperature, and very low cloud point and pour point values highlight its possibilities as an additive in D/ACE/SVO triple blends. Moreover, ACE can be produced through a renewable biotechnological process, an acetone–butanol–ethanol (ABE) fermentation from cellulosic biomass. The SVOs tested were castor oil (CO), which is not suitable for human consumption, and sunflower oil (SO), used as a standard reference for waste cooking oil. The viscosity measurement of the ACE/SVO double blend was considered crucial to choose the optimum proportion, which better fulfilled the specifications established by European standard EN 590. Moreover, some of the most significant physicochemical properties of D/ACE/SVO triple blends, such as kinematic viscosity, cloud point, pour point, and calorific value, were determined to assess their suitability as fuels. The blends were evaluated in a conventional diesel generator through the study of the following parameters: engine power, smoke emissions, and fuel consumption. Despite the low calorific value of ACE limits its ratio in the mixtures due to engine knocking problems, the experimental results reveal an excellent performance for the blends containing up to 16-18% of ACE and 22-24% of SVO. These blends produce similar engine power as to fossil diesel, but with slightly higher fuel consumption. Considerable reductions in emissions of air pollutants, as well as excellent cold flow properties are also obtained with these triple blends. In summary, the use of these biofuels could achieve a substitution of fossil diesel up to 40%, independently on the SVO employed.

## 1. Introduction

In accordance with the processes initiated in the last decade by many countries to reduce anthropogenic greenhouse gas (GHG) emissions [1], and despite the increased effort in the introduction of vehicles that incorporate electric or hydrogen engines, the gradual replacement of fossil fuels by biofuels is revealed as an imperative requirement, in order to accomplish the objectives set by international agreements as well as to ensure energy security [2,3].

Straight vegetable oils have become a potential alternative to attain a viable energy transition since they can be obtained from living plant sources, making them renewable and easily available biofuels. However, their high viscosity generates poor fuel atomization, leading to carbon deposition on the injector as a consequence of an incomplete combustion. Most vegetable oils exhibit kinematic viscosity values from 10 to 17 times greater than diesel fuel, even far superior in the case of castor oil, which exhibits a viscosity value of 226.2 centistokes (cSt). For this reason, engines of current vehicle fleets (more than one billion) cannot directly operate on triglycerides as drop-in biofuel. Thus, vegetable oils require previous treatments to reduce their viscosity values to fulfil the limits established by the European Normative for being used in compression–ignition (C.I.) engines. In addition to this, other drawbacks of straight vegetable oils (SVOs) as drop-in biofuel are lower volatility, poor cold flow properties, as well as polyunsaturated character. To overcome these problems, many researchers have developed different methods to transform these SVOs, such as pyrolysis, micro-emulsification, dilution, and transesterification [4].

The most widely used process to improve the fuel properties of triglycerides has been the transesterification reaction of the oil with an alcohol (usually methanol or ethanol) in the presence of alkaline catalysts, either homogeneous or heterogeneous, which gives rise to a mixture of mono-alkyl esters of long chain fatty acids, known as biodiesel. Alternatively, the alcoholysis of vegetable oils have been extensively studied through enzymatic catalysis in order to obtain greener fuel [5,6]. Either pure biodiesel or biodiesel-fossil diesel blends can exhibit adequate viscosity values, providing similar engine performance to that shown for the fossil diesel, even with lower emission levels [7]. Nevertheless, the glycerol obtained as a by-product in the synthesis of the biodiesel (at least 10% by weight of the starting raw material) has been recognized as one of the causes of the economic infeasibility of this process [8]. Glycerol must be separated from biodiesel because it can polymerize at the high temperatures reached in the engine. Furthermore, glycerol can promote the formation of acrolein, which is toxic at these temperatures. Therefore, glycerol involves not only the management of huge quantities of this by-product that currently does not find a market outlet, but also causes the contamination of biodiesel, which must be purified to eliminate glycerol up to maximum limits established by European Normative (EN) 14214 (<0.25 wt.%) [9].

Since biodiesel production is not economically viable, a great delay in the process of replacing fossil diesel by biofuels occurs at the present time. Numerous investigations have focused on the glycerol valorization, since it can transform in plenty of added-value products [10,11]. In addition, several alternatives to convert vegetable oils into high quality biodiesel-like biofuels, which avoid the glycerol generation, have been developed, such as Gliperol, DMC-Biod, or Ecodiesel. Likewise, another renewable diesel fuel, known as “green diesel”, has been produced by treatment of vegetable oils (cracking, pyrolysis, hydrodeoxygenation, and hydrotreating), exhibiting very close fuel characteristics to those of fossil diesel [12].

In this context, a new methodology to obtain drop-in biofuels able to work on the current C.I. diesel engines is still under study. Unlike the strategies based in chemical processes to reduce the viscosity of vegetable oils, this new strategy aims to reduce the high viscosity of SVOs by mixing them with low viscosity solvents (LVS) in the right proportions to get adequate values, according to EN 590 standard. In this way, the costs associated with oil transformation can be removed and above-mentioned limitations about direct use of them can be controlled. Despite that the majority of investigations have focused on the direct use of SVOs and their binary blends with fossil diesel, an intensive study about the performance and emissions of C.I. engines fueled with blends of SVO and different LVS can also be found in the literature. Thus, several alcohol-based fuels, such as ethanol or butanol [13,14,15,16], and plant-based light biofuels, such as eucalyptus oil and pine oil [17,18,19], have been tested as lower viscosity and lower calorific value (LVLC) fuels to reduce the high viscosity in blends [20]. This methodology has been also extended to gasoline, a non-renewable fuel with lower viscosity than conventional fossil diesel [21,22]. The diesel/gasoline/SVO blends exhibit a similar performance than diesel, but with a significant improvement in the biofuel properties, especially for its use in cold weather. Very recently, diethyl ether (DEE) has been applied as a LVLC solvent in blends with vegetable oils and diesel. The incorporation of DEE in the blends not only gives rise to good results, in term of engine power and emission reductions, but also leads to improvement in the cold flow behavior of fuel [23,24].

Alongside the abovementioned fuel additives, acetone is situated as an attractive oil solvent and a promising alternative fuel to fossil diesel. Although acetone (ACE) has usually been produced from fossil resources, it can also be obtained from renewable resources, either from ethanol [25] or from cellulose through a typical acetone–butanol–ethanol (ABE) fermentation process for bio-butanol production, which produces an acetone-butanol–ethanol mixture at a volumetric ratio of 3:6:1 [26]. In addition to its renewable nature and low kinematic viscosity, the ACE molecule has several favorable properties to be used as an alternative biofuel: its high oxygen content is beneficial to improve combustion quality, its high miscibility in vegetable oils and fossil diesel allows mixtures in any proportion, and the very low values of cloud point and pour point improve cold flow properties, making biofuel suitable for working in cold climates. Thus, a reciprocal benefit can be obtained by using ACE in blends with vegetable oils. On one hand, while acetone is able to reduce the viscosity of oils, the very high flash point temperatures of SVOs could compensate for the low flash pointof acetone, making the fuel safer for handling and transportation. On the other hand, the SVOs increase the lubricity of the blends. However, we reported in previous studies that the low calorific power of some LVLCs, such as ethanol and 2-propanol (27 and 33 MJ/kg, respectively), constitutes the greatest limitation for its use in double blends with oils [27]. A very similar behavior was observed for diethyl ether (34 MJ/kg) in blends with vegetable oils and diesel [24]. Therefore, the low calorific value of ACE (26.2 MJ/kg), which is comparable to that of the compounds previously studied (27–34 MJ/kg), could limit its proportion in blends with SVO and diesel to operate in today’s internal combustion diesel engines.

ACE has been used in mixtures with biodiesel to reduce pollutant emission and to enhance its flow properties at low temperatures [28,29,30]. Moreover, ternary blends, such as acetone/bioethanol/diesel, have been investigated in diesel engines [31,32]. On the other hand, this compound has been described as an effective fuel oxygenated additive being part of the ABE mixture along with diesel [33]. In fact, it was found that the high volatility of ACE makes it a combustion improver. Thus, diesel blended with acetone could significantly improve combustion properties of diesel and decrease soot emissions [34,35]. In view of the excellent effect of ACE blending with biodiesel and diesel, the application of acetone as a LVLC solvent of pure vegetable oils could be considered as an appropriate candidate to balance the viscosity of the blends. In addition, ACE can increase the engine fuel combustion efficiency, lessening the emission of polluting particles. In this way, a high rate of fossil diesel replacement could be attained, generating a viable biofuel, both from a technical and economic point of view.

Herein, we proposed several fuels, formulated with two different non-edible vegetable oils, sunflower waste cooking oil and castor oil. The lower environmental impact, along with sustainability and a reasonable price of these oils make them suitable candidates as diesel substitute biofuels. Both vegetable oils are considered as second-generation biofuels since they do not compete with other existing agricultural crops because they are not destined to food uses. Hence, the use of these oils avoids issues related to deforestation, global warming, and threats to biodiversity, which are usually associated with the use of edible oils. On one hand, castor is easily grown and resistant to drought. It requires very minimum fertilizer and irrigation, so that the cost of plantation is lower than the plantation cost for edible oil crops. Moreover, castor oil is currently the only inedible vegetable oil available on an industrial scale (about 220.000 tons/year). On the other hand, waste edible oils are generated in large amounts annually, so that waste cooking sunflower oil is readily available. In addition, its use as biofuel could cope with the disposal problem of this waste. Waste edible oils are generally cheaper than fresh edible oil as the costs associated with the collection and purifying processes are removed [36]. In order to obtain accurate data, sunflower oil has been selected as a standard reference of waste cooking oils. In this way, the problems associated with the reproducibility of results are solved, since waste cooking oils can be obtained from different sources.

According to the arguments previously exposed, the main purpose of this study is to investigate the influence of acetone on the performance and emission characteristics of a diesel engine operating with triple blends (diesel/acetone/straight vegetable oil). Likewise, some of the most determining properties of fuels, e.g., calorific value, cloud point, pour point, and kinematic viscosity, were determined. Then, the blends used as fuels containing 80% fossil diesel (D) and 20% SVO/ACE (B20), 60% D and 40% SVO/ACE (B40), 40% D and 60% SVO/ACE (B60), 20% D and 80% SVO/ACE (B80) and 100% SVO/ACE (B100), were tested in a conventional diesel engine, and compared to fossil diesel, studying relevant parameters, such as engine performance, fuel consumption, and soot emissions. Based on physical–chemical properties, and behavior of these mixtures in the engine, the impact of acetone as an alternative biofuel was evaluated.

## 2. Results and Discussion

### 2.1. Fuel Properties of ACE/SVO Double Blends and D/ACE/SVO Triple Blends

The kinematic viscosity values of acetone/sunflower oil and acetone/castor oil blends are shown in Table 1. According to the results, a reduction in the viscosity values of the blends was obtained when the concentration of ACE in the blend increases, as expected. It is interesting to remark the capability of ACE to reduce the viscosity of oils, mainly castor oil. In fact, with 20% of ACE in the blend, a reduction of the kinematic viscosity of sunflower oil from 37.8 to 11.46 cSt was obtained. This reduction was even higher in the case of castor oil, from 226.20 to 25.42 cSt. Among the different ACE/SVO blends tested, it was found that the blends that fulfil with the international fuel standards (2.0–4–5 cSt) are the ACE/ sunflower oil (SO) 40/60 and the ACE/ castor oil (CO) 45/55.

**Figure 1:**
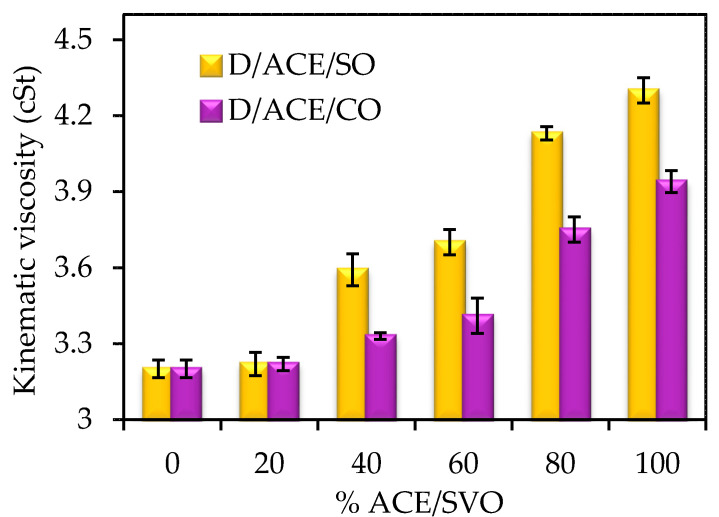
Kinematic viscosity values at 40 °C of diesel/acetone/sunflower oil and diesel/acetone/castor oil triple blends. The measure error is represented as standard deviation, using error bars.

Once the optimal percentage of acetone in double mixtures is known (40% in ACE/SO blend and 45% in ACE/CO blend), several triple mixtures have been prepared by addition of fossil diesel in proportions which vary from 20% to 80% by volume. The kinematic viscosity values of these triple blends were also determined, Figure 1. As can be seen, the addition of ACE/SVO biofuel to the blend, from B20 to B80, promotes an increase in the viscosity value of the blend, which was expected, taking into account that the viscosity of the B100 biofuels (between 4 and 4.3 cSt) was slightly higher than that of diesel (3.20 cSt). In any case, the viscosities of fuel blends vary in the range of 3.2–4.3 cSt and, therefore, they comply with European regulations EN 590 to be employed in diesel engines, which establish that viscosity at 40 °C must be in the range of 2.0–4.5 cSt.

The cloud point, pour point and calorific value of the proposed triple blends are collected in Table 2 (blends with SO) and Table 3 (blends with CO). The positive influence of acetone addition on the cold flow properties of triple blends is clearly revealed with both vegetable oils. Indeed, for the blends containing castor oil, the addition of 9% of ACE (B20 blend) is enough to reduce both, the pour point (PP) (from −16.0 to −23.5 °C) and the cloud point (CP) (from −6.0 to −17.0 °C). Likewise, in the blends containing sunflower oil, the incorporation of 8% of ACE to the blend reduce the CP and PP 9 and 5.5 °C, respectively. Therefore, the best CP and PP values are obtained with B40 blend, composed by 18% of ACE, 22% of castor oil, and 60% of diesel, resulting in a CP value of −18.0 °C and a PP value of −25.6 °C. A milder decrease in CP and PP temperatures is achieved in blends containing sunflower oil. In this case, the best behavior is reached adding 16% of ACE and 24% of SO, which exhibits a CP of −16.8 °C and a PP of −22.6 °C. The improvement of flow properties at lower temperatures observed for the analyzed blends could be explained by the fact that the incorporation of acetone either reduces the size or inhibits the formation of the wax crystallites formed upon cooling the fuel. It can be confirmed that the use of ACE as an additive promotes significant improvement, with regard to fossil diesel, on the cold flow properties of all the blends studied, maintaining appropriate viscosity values to be used as a biofuel in C.I. engines. In this way, ACE lets overcome poor cold flow properties of biodiesel, one of the major challenges to improve its use as an alternative to fossil diesel in current engines.

Regarding to the calorific values of triple blends (Table 2 and Table 3), the higher the percentage of acetone in blends, the lower the calorific value. Calorific values of samples, from B100 to B20, range between 35.0 and 41.4 MJ/kg, and they are approximately 3–18% lower than those of diesel (B0). As can be seen, the results were very similar, independently on the SVO employed, confirming the fact that both vegetable oils exhibit very similar calorific power. The best results were obtained on B20 triple blends, exhibiting the highest calorific value, 41.4 MJ/kg in the blend containing sunflower oil and 41.2 MJ/kg in the blend containing castor oil.

### 2.2. Mechanical Performance of a Diesel Engine Operating as Electric Generator

**Figure 2:**
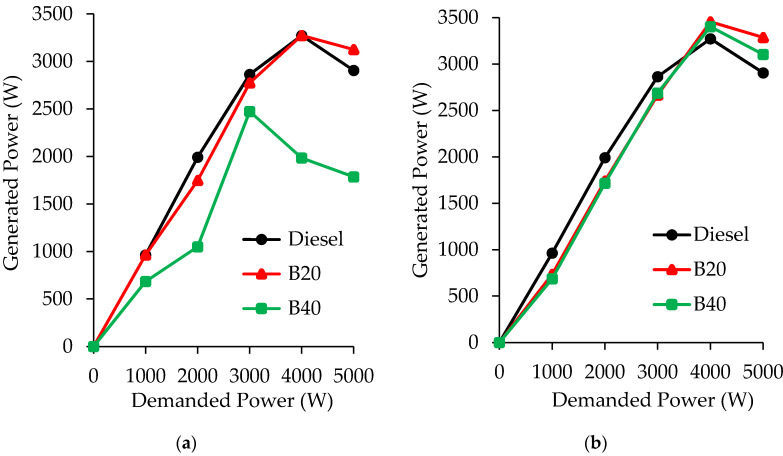
Generated power (W) at different demanded power values (1–5 kW) for: (**a**) diesel/acetone/sunflower oil; (**b**) diesel/acetone/castor oil blends. The errors in measurements are less than 3%.

The blends selected as biofuels, based on the study of their physical and chemical properties have been tested in a C.I. engine to determine the optimal proportion D/ACE/SVO that leads to the most adequate engine performance. The engine tests were carried out at different powers demanded, 0, 1, 2, 3, 4, and 5 kW. The results obtained with the blends D/ACE/SO are plotted in Figure 2a, and those corresponding to the D/ACE/CO blends are plotted in Figure 2b. In addition, the engine tests were also carried out with fossil diesel for comparison purposes. Overall, the power output increased as the power supplied also increased from 0 to 4 kW. A higher increase in the power supply (5 kW) promotes a slight decrease in power engine. It is noteworthy that the power generated by the engine with the tested blends is very similar and even higher to that obtained with diesel, mainly at the highest demanded power values (4 and 5 kW). This behavior keeps up for biofuels composed by a maximum percentage 16–18% of acetone, i.e., for B20 and B40. However, higher concentrations of both SVO and ACE (B60, B80, and B100 blends) led to knocking problems that prevent the proper running of the engine. This fact could be explained by the reduction in the energy content of the blends, as a consequence of the low calorific value exhibited by acetone, confirming the results obtained in previous investigations with triple blends containing ethanol [27] or diethyl ether [24] as LVLCs. If both vegetable oils are compared, it can be observed that blends composed by castor oil enable higher power output than blends with sunflower oil, especially in B40 blends. Since calorific value of sunflower oil is very close to that of castor oil (Table 2 and Table 3), the best behavior of castor oil could be ascribed to its higher cetane number value. Notwithstanding, the influence of other fuel parameters on engine performance cannot be ruled out.

### 2.3. Smoke Emissions: Opacity

**Figure 3:**
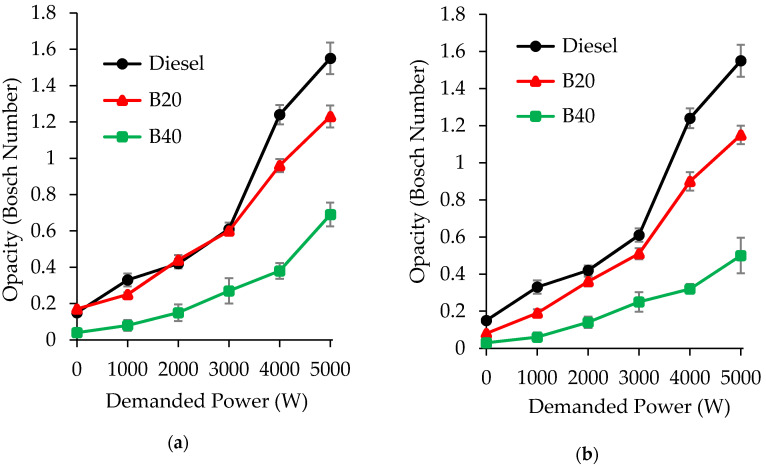
Smoke emissions (Bosch number) at different demanded power values (1–5 kW) for: (**a**) diesel/acetone/sunflower oil; (**b**) diesel/acetone/castor oil blends. The measure errors are represented as standard deviation using error bars.

Figure 3 shows the variation in the smoke emissions, expressed in Bosch number. In general, the D/ACE/SVO blends improve the smoke emissions, decreasing, noticeably, the opacity values, in comparison to conventional diesel. The highest reduction was obtained with B40 blends, independently on the SVO employed, i.e., with the highest proportion of renewable compounds in the blend. This fact can be explained, since ACE and SVO molecules are both rich in oxygen and their presence in the blends improve the combustion. Thus, it confirms the important role that oxygenated compounds play in diesel ignition and soot reduction. In particular, opacity values are about a 55–76% lower than those of diesel fuel for B40 D/ACE/SO (Figure 3a) and about 59–82% lower for B40 D/ACE/CO (Figure 3b). The best opacity values were then accomplished by the B40 blend containing castor oil, which provides a reduction of up to 82% at demanded power of 1kW. Comparing both tested oils, it is observed that opacity values obtained for biofuels with castor oils are slightly lower than those obtained with sunflower oil. It is known that the presence of unsaturation in fuels directly influences the formation of soot [37]. Hence, these differences could be justified by the presence of a major number of double bonds in the linoleic acid of sunflower oil, compared to those present in the ricinoleic acid of castor oil.

### 2.4. Fuel Consumption

**Figure 4:**
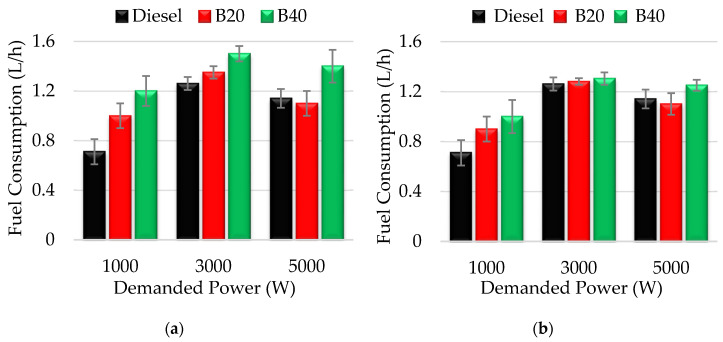
Fuel consumption values (L/h) at different demanded power values (1, 3, 5 kW) for: (**a**) diesel/acetone/sunflower oil; (**b**) diesel/acetone/castor oil blends. The measure errors are represented as standard deviation using error bars.

Another decisive parameter in the development of a new biofuel intended to be used as alternative to fossil diesel, is its consumption. Figure 4 shows the variation of consumed volume (in liters per hour) by the engine fueled with the different D/ACE/SVO triple blends, at low, medium, and high demands of power (1, 3 and 5 kW).

According to the results obtained, the presence of acetone and oil in blends usually generates greater consumption of fuel than conventional diesel. The cause of this increment in volume of fuel consumed is mainly attributed to the reduction on the energy content of the blends, since the incorporated compounds, specially ACE, exhibit a lower calorific value than diesel (Table 2 and Table 3). Therefore, when content of acetone in fuel increases, from B0 to B40, the volume consumed by engine is higher. Concretely, D/ACE/SO blends consumed up to 69% more than diesel at 1 kW of demanded power. On the contrary, the blends containing castor oil consume slightly less than their equivalents of SO, i.e., up to 41% more respect to diesel. The differences in consumed volume by the two types of fuels (with sunflower or castor oil) can be attributed to that the higher cetane number of castor oil decreases the ignition delay period, leading to a lower fuel consumption. For all the blends tested, the highest consumption is usually produced at lower power demands (1 kW). This can be due to the engine requires a greater volume of fuel to its initial start.

Be that as it may, and based on everything exposed above, it can be affirmed that both types of biofuels (D/ACE/SO and D/ACE/CO) are able to provide similar power values to those of fossil diesel or even superior in some cases (Figure 2), but in exchange, a higher volume of fuel is consumed.

## 3. Materials and Methods

Some of main physicochemical properties of diesel, sunflower oil, castor oil, and acetone are given in Table 4.

### 3.1. Preparation of Acetone/Vegetable Oil Double Blends and Diesel/Acetone/Vegetable Oil Triple Blends

Fossil diesel (D) was purchased from Repsol service station, sunflower oil (SO) was obtained from a local market, castor oil (CO) from Panreac Company (Castellar Del Vallès, Spain), and acetone (ACE) was acquired from Sigma-Aldrich Chemical Company (≥99.5% purity). Both vegetable oils (sunflower and castor oil) were mixed with ACE at different concentrations, so as to reduce the high viscosity of these oils and to obtain optimum ACE/SVO double blends in accordance with European diesel standard EN 590, which establishes that kinematic viscosity at 40 °C must be in the range of 2.0–4.5 cSt. The ideal ACE/SO and ACE/CO double blends were added to fossil diesel in several proportions by varying the volume percentage, from 20% to 80%. These mixtures are designated as B20, B40, B60, B80, and B100, where 100% fossil diesel is indicated as B0 and ACE/vegetable oil blend is referred as to B100. For example, B20 is composed of 20% ACE/vegetable oil and 80% diesel, Table 5 and Table 6. The components of all blends were manually mixed at room temperature. Furthermore, all components were completely miscible with fossil diesel, allowing the mixing of these in any proportion. All fuel triple blends, D/ACE/SO, and D/ACE/CO were evaluated in C.I. engine. Moreover, the ACE/SVO double blends (B100) were tested as biofuels.

### 3.2. Characterization of the Biofuel Blends

The properties of fuels have major role in safety, performance, and emissions of diesel engines and, thus, its study is essential to define fuel characteristics, which allow its commercialization. In the present investigation, some of the critical properties of blends, such as kinematic viscosity, pour point, cloud point, and calorific value have been determined either experimentally or using specific equations to predict them.

#### 3.2.1. Kinematic Viscosity

Viscosity values of the blends were measured with an Ostwald-Cannon-Fenske capillary viscometer (Proton Routine Viscometer 33200, size 150) at 40 °C, following the European standard EN ISO 3104 test method, by determining the time required for a known volume of liquid to pass under gravity between two marked points on the instrument, placed in an upright position. The kinematic viscosity (υ), which can be expressed in centistokes (cSt) or mm^2^/s, is obtained from the Equation (1):

(1) υ=C·t

where t is the flow time (expressed in seconds), and C is the calibration constant of the measurement system, specified by the manufacturer (in this case, 0.037150 (mm^2^/s)/s at 40 °C) [24]. The measures for viscosities were performed three times for each sample and the results are expressed as standard deviation.

#### 3.2.2. Pour Point and Cloud Point

Cloud point and pour point were measured following the standards EN 23015/ASTM D2500 for CP and ISO 3016/ASTM D97 for PP. The samples of different composition, were added to a flat-bottomed glass tube, which was tightly closed with a cork carrying a thermometer with a temperature measuring range of −36 to 120 °C. The tube was introduced in a digitally controlled temperature refrigerator for twenty-four hours. The samples were examined at intervals of 1 °C, until the appearance of turbidity at the bottom of the tube was observed, which is indicative that the CP temperature has been reached. After a progressive decrease in temperature, the samples were kept under observation until liquid ceased flow totally (i.e., liquid not showed any movement when tube was held in a horizontal position for 5 s). To the observed temperature are added 3 °C additional. This temperature is recorded as the pour point [24]. All values are the mean of duplicate determinations.

#### 3.2.3. Calorific Value

Calorific value (CV) or heat of combustion, commonly expressed in kJ/Kg, is defined as the quantity of heating energy released during complete combustion of a unit mass of the fuel. This property is considered as totally decisive to determine the suitability of a biofuel as an alternative to fossil diesel, since it has a direct effect on the power output of an engine. Moreover, this parameter provides an estimate of fuel consumption. In general, it is desirable that compounds added into the blend exhibit a higher calorific value than the diesel fuel [42]. The CV of a molecule is greater as its chain length is increased and unsaturation degree is lower. This property can be determined experimentally by a bomb calorimeter, but a theoretical value can be usually calculated, according to the volumetric concentration of each components in the blend, from the following equation:

(2) $$\mathrm{CV}=\sum_{i}\mathrm{CVi}\mathrm{Xi}$$

where CVi is the calorific value of each component and Xi is the percentage of every component [24].

### 3.3. Mechanical and Environmental Characterization of a Diesel Engine-Electrogenerator Set Fueled with Different Double and Triple Blends Biofuels

**Figure 5:**
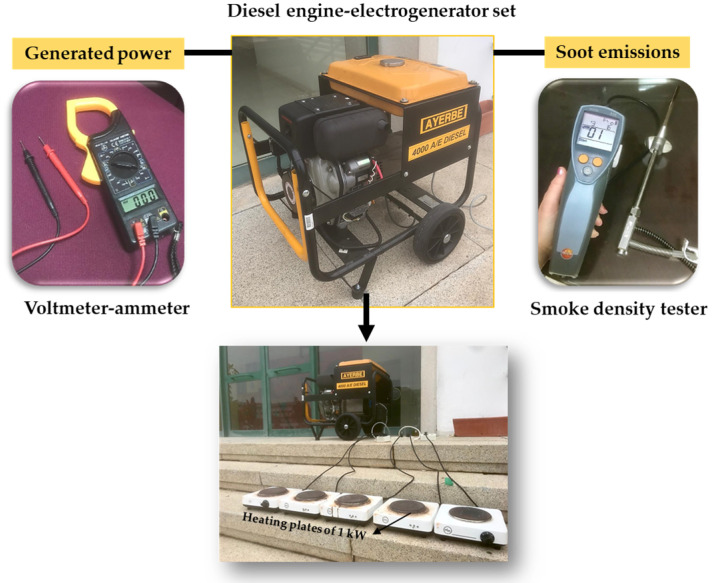
Schematic summary of mechanical and environmental characterization of diesel engine-electrogenerator set.

The energy performance and pollutants emissions have been analyzed following the experimental methodology previously described [22,24,27], on a diesel engine–electric generator set (Model: AYERBE AY- 4000 D, Vitoria, Spain), working at a rate of 3000 rpm with a power of 5 KVA and 230 V, for the generation of electricity. Different degrees of demand for electrical power are applied by connecting heating plates of 1000 watts each one (Figure 5). This diesel engine has operated at a constant rate of rotation of the crankshaft and torque, so that the different values of electrical power obtained are an exact consequence of the mechanical power obtained after the combustion of the corresponding biofuel. The experimental tests were carried out by supplying to the engine identical volume of the different biofuel’s blends. The electrical power (P) generated can be easily calculated from the product of the potential difference (or voltage) (V) and the electric current intensity (or amperage) (I), Equation (3), both obtained by means of a voltmeter ammeter, Figure 5.

P (watts) = V (volts) × I (amps)(3)

The fuel consumption of the diesel engine, fueled with the different biofuels studied, was calculated estimating the speed of consumption of the engine when it operates under a determined demand of electric power (1, 3, or 5 kW). Thus, the operation times are achieved by operating under the same fuel volume (0.5 L).

The contamination degree is evaluated from the opacity of the smoke generated in the combustion process, which is measured by the smoke opacity meters. The smoke opacity meters are instruments capable of measuring the optical properties of diesel exhaust. These instruments have been designed to quantify the visible black smoke emission making use of physical phenomena as the extinction of a light beam by scattering and absorption. There are two groups of instruments: opacity meters, which evaluate smoke in the exhaust gas, and smoke number meters, which optically evaluate soot collected on paper filters. The density gauge is a handheld instrument for determining the filter smoke number (FSN), the Bosch number, and the soot concentration of diesel engines. This instrument is composed of an optical sensor (photodiode) and a differential pressure sensor. The photodiode calculates the paper blackening based on the reflected light intensity by a white LED. The more soot is deposited on the filter paper, the less light is reflected. The probe volume determined by the differential pressure sensor is used to calculate the probe volume under reference conditions with the input height and the temperature measured by the instrument. This probe volume and the measured paper blackening are then used to calculate the FSN, soot concentration (mg/m3) or Bosch smoke number. Herein, the exhaust emissions were measured by a Bosch smoke meter or opacimeter-type smoke tester TESTO 338 density gauge, following the European Union (EU) Directive 2004/108/EC, at the operating conditions previously reported [24] (Figure 5). In this research, the generated smoke results are expressed as Bosch number, which is a standardized unit calculated from the level of soot on the paper (effective filter loading). The instrument evaluates smoke density in a scale from 0 to 2.5, where the value 0 represents total clarity on the paper and 2.5 is the value corresponding to 100% cloudy, as established by ASTM D 2156, Standard Test Method for Smoke Density in Flue Gases from Burning Distillate Fuels.

Each fuel test was repeated three times and the results are shown as average along with standard deviation, represented as error bars. All results obtained with the biofuels evaluated were compared with those obtained with conventional diesel.

## 4. Conclusions

The present study analyzed the feasibility of acetone as a solvent of vegetable oils, to be blended directly with diesel and form D/ACE/SVO triple blends, which can be used as fuel in diesel engines. The evaluation of the performance and combustion quality of a diesel engine was conducted through the study of three crucial parameters in engine behavior: electrical efficiency, fuel consumption, and exhaust emissions. Engine tests were carried out with two types of fuel blends diesel/acetone/sunflower oil and diesel/acetone/castor oil, composed by different proportions of each compound, namely B20, B40, B60, B80, B100; results were compared to the reference fossil diesel fuel. Moreover, the mixtures were subjected to the study of some of the most influential physical–chemical properties in their performance as fuels. The experimental results revealed the following conclusions:

1. All D/ACE/SVO triple blends proposed fulfil necessary requirements of kinematic viscosity for being used in current diesel engines, since viscosity values of blends are found within limits established by the European diesel standard EN 590. Further, cold flow properties are significantly improved as compared to diesel, by incorporation of acetone, making engines run better in cold weather.
2. The effect of low calorific value of acetone on the energy content of the blends has been reflected in performance of the diesel engine, which did not work properly with a percentage of acetone greater than 16–18% (B60-B100 blends). Despite the reduction in energetic content of blends, very good results of generated power have been achieved with B20 and B40 blends, similar, and even better, compared to diesel fuel, primarily when castor oil was employed.
3. A remarkable decrease in smoke emissions has been accomplished for all fuels tested. In particular, very low opacity levels are obtained for B40 blends due to their major oxygen content, which acts as an improver of combustion reaction. Besides, biofuels composed by castor oil rather than sunflower oil have an effect slightly stronger on minimizing soot formation.
4. The blends studied gave rise to a higher fuel consumption than conventional diesel due to acetone’s lower calorific value.
5. The best results, in term of generated power, reduction in exhaust emissions, and fuel consumption was reached using the blend B20; constituted by 80% diesel, 9% acetone, and 11% castor oil.
6. By using these triple blends, a replacement of fossil diesel up to 40% can be achieved, preventing any major modifications in engines of current vehicle fleets. Moreover, the possibility of synthesis of ACE through the ABE fermentation process, makes the substitution completely renewable.
7. The use of acetone allows the direct application of vegetable oils as biofuels in current diesel engines, avoiding energetic and economic costs associated with previous treatments.
8. Ultimately, it can be concluded that ACE could be a good alternative biofuel, as a component of blends with diesel and vegetable oils, due to its green-friendly production process, the potential to improve combustion efficiency, and lower pollutant emissions. The use of these biofuels could provide a meaningful advance in the replacement process of fossil sources, promoting independence from imported petroleum at the same time that greenhouse gases are minimized.

## Figures and Tables

**Table 1:** Kinematic viscosity values at 40 °C of acetone (ACE)/ sunflower oil (SO) and ACE/ castor oil (CO) double blends with different proportions of acetone; cSt, centistokes.

Property	Blend	Acetone (% by Volume)						
		0	20	40	45	50	60	100
**Kinematic viscosity (cSt)**	ACE/SO	37.80 ± 0.46	11.46 ± 0.26	4.30 ± 0.06	3.70 ± 0.10	3.10 ± 0.03	2.27 ± 0.06	0.35 ± 0.01
	ACE/CO	226.20 ± 0.55	25.42 ± 0.31	5.14 ± 0.09	3.94 ± 0.12	2.95 ± 0.05	2.0 ± 0.10	0.35 ± 0.01

**Table 2:** Cloud point, pour point and calorific value of diesel/acetone/sunflower oil blends, obtained by adding different amounts of fossil diesel to acetone/sunflower oil double mixture, containing 40% acetone. Errors are always calculated from the average of three measurements.

Nomenclature (% Renewable)	D/ACE/SO	Cloud Point (°C)	Pour Point (°C)	Calorific Value (MJ/kg)
B0	100/0/0	−6.0 ± 1.0	−16.0 ± 1.2	42.8
B20	80/8/12	−15.0 ± 1.1	−21.5 ± 1.0	41.4
B40	60/16/24	−16.8 ± 1.2	−22.6 ± 1.0	39.9
B60	40/24/36	−15.2 ± 0.9	−21.3 ± 1.3	38.5
B80	20/32/48	−14.2 ± 0.9	−20.5 ± 0.8	37.0
B100	0/40/60	−14.8± 1.0	−21.0 ± 1.0	35.6

Abbreviations: B0, 100% diesel; B20, 80% diesel + 20% ACE/SVO blend; B40, 60% diesel + 40% ACE/SVO blend; B60, 40% diesel + 60% ACE/SVO blend; B80, 20% diesel + 80% ACE/SVO blend; B100, 100% ACE/SVO blend.

**Table 3:** Cloud point, pour point, and calorific value of diesel/acetone/castor oil blends, obtained by adding different amounts of fossil diesel to acetone/castor oil double mixture, containing 45% acetone. Errors are always calculated from the average of three measurements.

Nomenclature (% Renewable)	D/ACE/CO	Cloud Point (°C)	Pour Point (°C)	Calorific Value (MJ/kg)
B0	100/0/0	−6.0 ± 1.0	−16 ± 1.2	42.8
B20	80/9/11	−17.0 ± 1.1	−23.5 ± 1.0	41.2
B40	60/18/22	−18.0 ± 0.7	−25.6 ± 1.3	39.7
B60	40/27/33	−17.3 ± 1.2	−23.1 ± 1.2	38.1
B80	20/36/44	−16.0 ± 0.9	−21.5 ± 1.0	36.6
B100	0/45/55	−18.0 ± 1.0	−22.1 ± 0.8	35.0

**Table 4:** Properties of diesel, sunflower oil, castor oil, and acetone [38,39,40]. Kinematic viscosity values were experimentally determined in the present work.

Property	Diesel	Sunflower Oil	Castor Oil	Acetone
Density at 15 °C (kg/m^3^)	830	920	962	791
Kinematic viscosity at 40 °C (cSt)	3.2	37.8	226.2	0.35
Calorific value (MJ/kg)	42.8	39.6	39.5	29.6
Flash point (°C)	66	220	228	−20
Auto-ignition temperature (°C)	250	316	448	560
Cetane number	50	37	40	- ^1^

^1^ The exact value is not found in the literature, but its saturated vapor pressure much higher and boiling point much lower than that of diesel, allow to predict a high cetane number for acetone [41].

**Table 5:** Diesel/acetone/sunflower oil blends containing 40% acetone.

Nomenclature	D/ACE/SO	% Diesel	% Acetone	% Sunflower Oil
B0	100/0/0	100	0	0
B20	80/8/12	80	8	12
B40	60/16/24	60	16	24
B60	40/24/36	40	24	36
B80	20/32/48	20	32	48
B100	0/40/60	0	40	60

**Table 6:** Diesel/acetone/castor oil blends containing 45% acetone.

Nomenclature	D/ACE/CO	% Diesel	% Acetone	% Castor Oil
B0	100/0/0	100	0	0
B20	80/9/11	80	9	11
B40	60/18/22	60	18	22
B60	40/27/33	40	27	33
B80	20/36/44	20	36	44
B100	0/45/55	0	45	55

## Nomenclature

ACE	Acetone
ASTM	American society for testing and materials
B0	100% diesel
B20	80% diesel + 20% ACE/SVO blend
B40	60% diesel + 40% ACE/SVO blend
B60	40% diesel + 60% ACE/SVO blend
B80	20% diesel + 80% ACE/SVO blend
B100	100% ACE/SVO blend
CI	Compression ignition
CO	Castor oil
CP	Cloud point
cSt	Centistokes
CV	Calorific Value
D	Diesel
FSN	Filter smoke number
ISO	International Standards Organization
LVLC	Lower viscosity and lower calorific value
LVS	Low viscosity solvent
PP	Pour point
rpm	Round per minute (min^−1^)
SO	Sunflower oil
SVO	Straight vegetable oils
W	Watts

## Symbols

A	Amperage (amps)
C	Calibration constant (mm2/s)/s
P	Electrical power (watts)
t	Flow time (s)
V	Voltage (volts)
υ	Viscosity (centistokes)
